# Congruent relations between perceived neighbourhood social cohesion and depressive symptoms among older European adults: An East-West analysis

**DOI:** 10.1016/j.socscimed.2019.112454

**Published:** 2019-09

**Authors:** Milagros Ruiz, Sofia Malyutina, Andrzej Pajak, Magdalena Kozela, Ruzena Kubinova, Martin Bobak

**Affiliations:** a Research Department of Epidemiology and Public Health, University College London, 1-19 Torrington Place, London, WC1E 7HB, United Kingdom; b Faculty of Physical Education and Sport, Charles University, José Martího 31, Prague 6, 162 52, Czech Republic; c Research Institute of Internal and Preventive Medicine, Branch of the Institute of Cytology and Genetics, SB RAS, Prospekt Lavrentyeva 10, 630090, Novosibirsk, Russia; d Novosibirsk State Medical University, Borisa Bogatkova 175/1, 630089, Novosibirsk, Russia; e Institute of Public Health, Faculty of Health Sciences, Jagiellonian University Medical College, Grzegórzecka 20, 31-531, Krakow, Poland; f Centre for Environmental Health Monitoring, National Institute of Public Health, Šrobárova 48, 10042, Prague, Czech Republic

**Keywords:** Central and Eastern Europe, Cohort study, Comparative study, Depressive symptoms, Depression, England, Neighbourhoods, Social cohesion

## Abstract

**Rationale:**

Two gaps in the literature arise on the relationship between social cohesion and depressive disorders. Firstly, there is a lack of studies comparing countries with diverse communal bonds and population-level differences in depression. Secondly, most work on explanatory mechanisms has overwhelmingly focussed on social network and social support pathways.

**Objectives:**

We compared the prospective association between perceived neighbourhood social cohesion and depressive symptoms among older adults in England, the Czech Republic, Poland and Russia; and examined whether psychological and health behavioural pathways mediated this association.

**Methods:**

Harmonized data on 26,081 adults from the English Longitudinal Study of Ageing (ELSA), and the Health, Alcohol and Psychosocial factors In Eastern Europe (HAPIEE) studies were analysed. Prospective associations between perceived neighbourhood social cohesion at baseline and depressive symptoms at follow-up were assessed using multivariable negative binomial regression. The psychological (through control of life, and control at home) and health behavioural (through smoking and drinking) pathways were tested using path analysis.

**Results:**

Low cohesion predicted a higher number of depressive symptoms at follow-up among English (b = 0.106, p = 0.001), Czech (b=0.203, p < 0.001), Polish (0.115, p < 0.001) and Russian adults (b = 0.087, p < 0.001). Indirect effects via psychological mechanisms were strong and explained 64% (Poland), 82% (Russia), 84% (England) and 95% (Czech Republic) of the total indirect effects from low cohesion to elevated symptoms in these populations. Indirect effects via health behaviours were much weaker by comparison.

**Conclusions:**

Prospective associations between low social cohesion and increased depressive symptoms were largely congruent among older adults from England and three Central and Eastern European countries. These associations operated via a psychological, but not a health behavioural, pathway among ageing adults living in diverse parts of Europe.

## Highlights

• Social cohesion protects against depressive symptoms in older European adults.
• The association operates via psychological, but not health behavioural, mechanisms.
• Findings were highly consistent between English, Czech, Polish and Russian adults.

## 1 Introduction

Adults face a greater incidence of depressive disorders in later life; due to the onset of less severe forms of depression, such as dysthymia, minor depression and unspecified depressive disorder (Haigh et al., 2018). Older adults with these disorders endure social, physical and role functioning impairments (Haigh et al., 2018) and mortality rates (Cuijpers and Smit, 2002; Penninx et al., 1999) similar to those with major depression. All manifestations of late life depressive disorders, therefore, pose considerable public health consequences for a rapidly ageing Europe.

Cross-national studies suggest that depressive symptomatology is more pervasive among older adults living in Central/Eastern and Southern countries than North-western European countries (Castro-Costa et al., 2007; Kok et al., 2012). These country differences emphasize macro-level determinants that may trigger proximal causes of depressive disorders; such as the upstream influence of social cohesion (De Silva et al., 2005; Ehsan and De Silva, 2015; Julien et al., 2012). A cultural dimension of the macro-social environment (Berkman et al., 2000), social cohesion refers to the presence of communal bonds characterised by altruism, reciprocity, and shared norms and values (Kawachi and Berkman, 2000). Country differences in social cohesion possess a striking similarity to country differences in depressive symptomatology, as social cohesion levels are noticeably lower in Central/Eastern and Southern countries than elsewhere in Europe (Anderson and Unzicker, 2014; Kääriäinen and Lehtonen, 2006; Poortinga, 2006). Given this clustering within particular European countries, cross-cultural work that compares the association between social cohesion and depressive symptomatology in countries with dissimilar population-level distributions are particularly valuable (Berkman et al., 2000).

Despite the wealth of evidence relating social cohesion to depressive symptoms and disorders, two gaps emerge in the literature. First, most European studies are limited to the UK and North-western Europe (De Silva et al., 2005; Ehsan and De Silva, 2015; Julien et al., 2012), which hinder cross-cultural comparisons that reflect the region's diversity. Second, few studies have investigated the potential mechanisms at play (Julien et al., 2012), and existing explorations have overwhelmingly focussed on social networks and social support (Choi et al., 2015; Stafford et al., 2011) in neglect of other pathways (Berkman et al., 2000; Blair et al., 2014). While it has been theorised that social cohesion may reduce the risk of depressive disorders by eliciting positive psychological states and protecting against daily stressors, and by discouraging harmful health behaviours through the regulation of social norms (Berkman et al., 2000; Blair et al., 2014; Kawachi and Berkman, 2001), these pathways remain unexplored.

This study compares the prospective association between perceived neighbourhood social cohesion and depressive symptoms among older adults in England and the Czech Republic, Russia and Poland; and examines whether psychological and health behavioural pathways mediate this association.

## 2 Methods

### 2.1 Participants

We employed data from two population-based European prospective cohort studies of ageing: The English Longitudinal Study of Ageing (ELSA) (England), and the Health, Alcohol and Psychosocial factors In Eastern Europe (HAPIEE) study (Czech Republic, Russia and Poland). Both studies were designed to monitor the long-term health of community-dwelling adults aged 50+ (ELSA) and 45–69 (HAPIEE). Participants were drawn from a sampling frame generalizable to the English population in ELSA, and randomly selected from population registers in six medium-sized Czech cities, Krakow (Poland) and Novosibirsk (Russia) in HAPIEE. Baseline assessment of the original ELSA (n = 11,391) and HAPIEE cohort samples in the Czech Republic (n = 8857), Poland (n = 10,728) and Russia (n = 9360), respectively, took place in 2002/3 and 2002/5. These sample sizes corresponded to individual response rates of 67% in ELSA, 55% in HAPIEE-Czech Republic and 61% in HAPIEE-Poland and HAPIEE-Russia (Peasey et al., 2006; Steptoe et al., 2013). The first follow-up assessments were conducted in 2004/5 and 2006/8, in turn for ELSA and HAPIEE; whereby 77% (n = 8780), 59% (n = 5097), 62% (n = 6721) and 66% (n = 6417) of the original English, Czech, Polish and Russian cohort samples were successfully re-examined in that order (Horvat et al., 2016; Steptoe et al., 2013). Data from these first two assessments were retrospectively harmonised for the present analysis. Full details on ethical approval have been previously reported (Peasey et al., 2006; Steptoe et al., 2013).

### 2.2 Measures

#### 2.2.1 Depressive symptoms

The Center for Epidemiological Depression (CES-D) scale was used to establish depressive symptoms in both studies. The CES-D scale is an internationally validated screening instrument that detects those who may be at high risk of depression or in need of specialist treatment among the general population, including older adults (Hertzog et al., 1990; Radloff, 1977). Originally developed in the English language; Czech, Russian and Polish language versions of the CES-D were subsequently validated for each respective country (Dershem et al., 1996; Dojka et al., 2003; Osecka, 1999).

The CES-D 8 and 10 item versions were implemented with yes/no response options at follow-up, respectively in ELSA and HAPIEE. For HAPIEE, the CES-D 8 version was simulated by eliminating two items on interpersonal relations that formed the CES-D 10. The CES-D 8 measured whether 8 symptoms on depressed affect, somatic and retarded activity and positive affect were experienced for ‘much of the time during the past week’ (Radloff, 1977; Steffick, 2000). Affirmative and negative responses to the first 6 and last 2 items, respectively, each counted as 1; and were summed to derive a score ranging from 0 to 8 for participants with data on at least 6 of the 8 items, as scoring guidelines require complete data on at least 75% of the CES-D scale (Radloff, 1977).

The prospective analysis accounted for baseline risk of probable depression in each study. ELSA participants with CES-D 8 scores of 3 were classified as probable cases. As HAPIEE employed the original CES-D scale (20 items measured using a 4-point Likert scale) at baseline, HAPIEE participants with CES-D 20 scores of 16 ≥ were categorised as probable cases. Each version-specific cut-off has been shown to sufficiently discriminate depressive disorders as those made by clinical diagnoses among older adults (Beekman et al., 1997; Turvey et al., 1999). As with the follow-up data, the classification of probable depression at baseline was made only on participants with sufficient CES-D data (i.e., those who responded to at least 6 of 8 items in ELSA, or 16 of 20 items in HAPIEE).

#### 2.2.2 Perceived neighbourhood social cohesion

Two similar items on perceived neighbourhood social cohesion (PSC) were collected at baseline. Items on interpersonal trust and help from neighbours were worded as follows: i) ‘Most people in this area can't be trusted’/‘Most people in this area can be trusted,’ and ‘Is there trust among people in your area of residence?’ and ii) ‘If you were in trouble, there is nobody in this area who would help you’/‘If you were in trouble, there are lots of people in this area who would help you,’ and ‘Would your neighbours help you if you needed it?’ respectively in ELSA and HAPIEE. Each set of opposing statements in ELSA was rated on a bipolar scale ranging 1–7. HAPIEE questions were answered on a unipolar scale ranging 1–5. PSC scores, derived by summing participant responses in ELSA (2–14), and HAPIEE (2–10), were negatively skewed in each country sample. Within HAPIEE, median scores were highest in Russia, followed by Poland and the Czech Republic. PSC scores were therefore harmonised by creating country-specific tertiles for ELSA: high (13–14), medium (11–12), low (2–10); HAPIEE-CZ: high (9–10), medium (7-8), low (2–6); HAPIEE-PO: high (9–10), medium (8), low (2–7) and HAPIEE-RU: high (10), medium (8–9), low (2–7).

#### 2.2.3 Covariates

Two self-efficacy measures at baseline comprised the psychological pathway. The degree of control over one's life and within one's household were assessed, respectively, by the subsequent statements, ‘In general, I feel that what happens in my life is often determined by factors beyond my control’ and ‘In general, at home I feel I have control over what happens in most situations,’ on a six-point Likert scale ranging from strongly agree (1) to strongly disagree (6). As 70% ≥ of participants ‘strongly, moderately and slighted agreed’ to having *low control over their life*, but ≤7% ‘strongly, moderately and slightly disagreed’ to having *high control within the home* in each study; the responses were dichotomised in order to compare high versus low levels of control that were appropriate for each distribution. Participants who responded ‘strongly agree’ to the first statement were considered as having low control of their life; and those who answered ‘strongly, moderately and slightly disagree, and slightly agree’ to the second statement were categorised as having low control at home.

The health behavioural pathway was tested using smoking and alcohol measures at baseline. Current smoking status was classified as never, former or current smokers in both studies. Drinking frequency categories of alcoholic beverages during the past year were made compatible in ELSA and HAPIEE by grouping data to a 5-point ordinal scale: i) Never, ii) Less than once a month, iii) Once or several times a month, iv) Once or twice a week and v) More than once or twice a week.

Age (years), gender, educational attainment, marital status, self-rated health and number of limitations in activities of daily living (ADLs) were selected as baseline covariates.

### 2.3 Analytic samples

Original cohort sample participants with sufficient CES-D data, according to CES-D scoring guidelines, at baseline and follow-up constituted the ELSA-EN (n = 8519), HAPIEE-CZ (n = 4908), HAPIEE-PO (n = 6474) and HAPIEE-RU (n = 6180) analytic samples. Exclusion criteria applied to form each analytic sample are depicted in Supplementary Figure 1.

### 2.4 Statistical analyses

#### 2.4.1 Multiple imputation

Study variables were complete for 42.8% (n = 3646) and 41.3% (n = 7259) of the ELSA and pooled HAPIEE analytic samples, respectively. Missing data were resolved by multiple imputation by chained equations which generated 20 imputed data sets for each sample using Stata V.15. As the analytic samples included participants with sufficient, yet incomplete, data on the CES-D scale at baseline and follow-up; the imputation addressed item non-response in order to calculate CES-D scores for all participants. As smoking status, drinking frequency, educational attainment, self-rated health and number of limitations in ADLs predicted attrition from baseline to follow-up in both studies; their inclusion in the imputation and analysis models helped adhere to the missing at random mechanism.

#### 2.4.2 Multivariable regression

Longitudinal associations between PSC and depressive symptoms were evaluated using multivariable negative binomial regression. Models were fitted in each country sample, and adjusted for the following explanatory covariates at baseline: age (years), gender, educational attainment, marital status, self-rated health, number of limitations in ADLs and probable depression. As two-way interaction terms between PSC and gender, and PSC and age group did not reach statistical significance; the analyses were not stratified on these characteristics.

#### 2.4.3 Path modelling

**Fig. 1:**
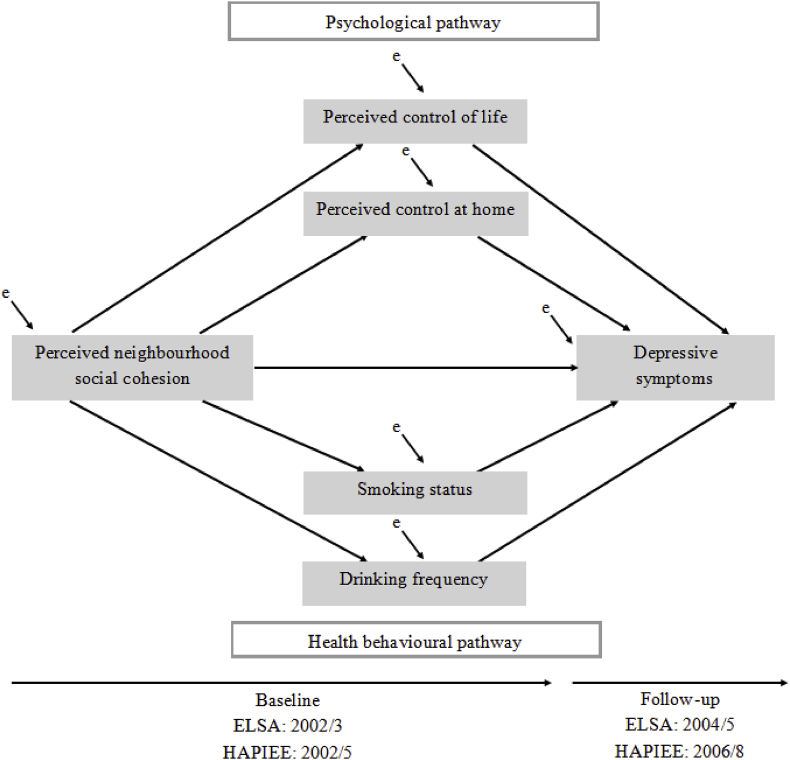
Path model of relations between perceived neighbourhood social cohesion and depressive symptoms, ELSA and HAPIEE. For simplicity, independent exogenous variables (age, gender, country (HAPIEE only), educational attainment, marital status, self-rated health, number of limitations in activities of daily living and probable depressive cases) of the model are not depicted in the figure. The exogenous variables are included as predictors of PSC and depressive symptoms.

Path modelling tested the hypothesised indirect effects of psychological and health behavioural pathways on the relations between PSC and depressive symptoms in each country sample, as diagrammed in Fig. 1. The unidirectional straight arrows indicate the paths that were estimated in the model, which presumes that the relations between PSC and depressive symptoms partially or fully operate via psychological and health behavioural pathways. These indirect effects are shown by the arrows from PSC to each pathway indicator, and in turn from each pathway indictor to depressive symptoms. Taken together, these measures are considered endogenous (shown as grey squares) because they are predicted by at least one variable in the model. Corresponding error terms (shown as small diagonal arrows) represent the variance of the endogenous variable that are not explained by its set of predictor(s) (Baron and Kenny, 1986; Ditlevsen et al., 2005; Hall and Sammons, 2013). For simplicity, Fig. 1 does not depict the model's independent exogenous variables, which are the explanatory covariates included in the multivariable regression.

The effect sizes and the direction of associations between PSC and depressive symptoms for each path are provided as unstandardized path coefficients (b). Paths pointing to the number of depressive symptoms were estimated using negative binomial regression. Unstandardized coefficients denote the difference in the expected log count of the number of depressive symptoms by each predictor. Paths pointing to each of the hypothesised mediators were estimated using logistic and ordered logistic regression. For smoking status, estimates for the former smoker category were retained in the model to better differentiate the behaviour over a binary classification of non-smokers and smokers, but not reported as an effect between PSC (at baseline) and former smoking (prior to baseline) does not meet the temporal precedence hypothesised by the model. Unstandardized logistic regression coefficients express the change in the predicted log odds or ordered log odds of being in the exposed group(s) for each categorical or ordered categorical measure for those with medium and low PSC, respectively.

The path models further demonstrate the extent to which the total effect between PSC and depressive symptoms is partitioned according to the relations hypothesised in Fig. 1. The path model measures: i) the direct effect from PSC to depressive symptoms, ii) the indirect effect from PSC to depressive symptoms via each pathway variable, iii) the total indirect effect, and iv) the total effect of PSC on depressive symptoms. The direct effect (i) of PSC on depressive symptoms is the portion of the model that does not operate through any of the pathway variables (i.e., the path coefficient from PSC to depressive symptoms). The indirect effect (ii) is the product of the path coefficients from PSC to a hypothesised mediator, and from that mediator to depressive symptoms. The total indirect effect (iii) is obtained by summing all indirect effects that are measured in the model. Lastly, the total effect (iv) is attained by summing the direct (i) and the total indirect (iii) effects (Alwin and Hauser, 1975). The decomposed effects denote the differences in the expected log count of the number of depressive symptoms that are observed by each portion of the model. All statistical analyses of the multiply imputed datasets were performed with robust maximum likelihood estimation using Mplus V.7. The ELSA-EN analyses included the non-response survey weight at baseline to ensure the analytic sample is representative of community dwelling adults aged 50 + in 2002/3.

#### 2.4.4 Sensitivity analyses

We performed three sets of sensitivity analyses. First, as the multivariable regression and path analyses adjusted for baseline depressive symptoms, we examined whether the presence of symptoms at baseline biased any of the longitudinal findings. Thus, we replicated the analyses among non-cases at baseline in each country sample: ELSA-EN (n = 6516), HAPIEE-CZ (n = 3868), HAPIEE-PO (n = 4649) and HAPIEE-RU (n = 4534). Second, given the differences in age profiles between ELSA-EN (50 + years) and the HAPIEE cohorts (45–69 years) at baseline, we repeated all ELSA analyses on a subset of participants aged 50–69 years (n = 5913) to determine whether the presence of participants aged 70 and older modified the main results in ELSA. Third, since drinking frequency over the past year was the only drinking measure available at baseline in ELSA, the path models relied on this indicator to test a health behavioural pathway across the four samples. For the purpose of considering other important aspects of drinking behaviour, we fitted two additional path models in the HAPIEE samples that tested alternate pathway effects via annual drinking volume and annual binge drinking frequency, in turn. Annual drinking volume (grams of ethanol) was classified using categories specific to men: i) 0, ii) 1–1,500, iii) 1501–4,000, iv) 4001–8000 and v) > 8000; and women: i) 0, ii) 1–250, iii) 251–500, iv) 501–1500 and v) > 1500 (Hu et al., 2015). Binge drinking episodes were defined as having 5 ≥ drinks in 1 day, which were grouped into: i) Never in the past year, ii) 1–5 times in the past year, iii) 6–12 times in the past year and iv) 2–3 times per month or more.

## 3 Results

### 3.1 Study characteristics

Table 1 describes the study characteristics in the four country samples. At follow-up, the mean number of depressive symptoms were higher in HAPIEE-RU (2.9) and HAPIEE-PO (2.2) than in ELSA-EN (1.6) and HAPIEE-CZ (1.1). As the range and distribution of PSC scores were dissimilar between countries, the ordered PSC scores fell more neatly into high, medium and low thirds of the population in HAPIEE-PO and HAPIEE-RU. Consequently, participants categorised as having low PSC were most frequent in ELSA-EN at 43%, and least common in HAPIEE-CZ at 20%. ELSA-EN participants, on average aged 64 years, were slightly older than HAPIEE participants aged 58 years. Women comprised a greater share of each sample by 2–5%. Levels of low control of life were similar between countries and ranged from 13% to 16%. Low control at home, however, was more widespread in the HAPIEE countries (17–21%) than in ELSA-EN (9%). Current smokers were more predominant in the HAPIEE samples, which coincided with the noticeably greater share of former smokers in ELSA-EN. Alcohol drinking frequencies were highest in ELSA-EN and lowest in HAPIEE-PO, as participants who drank once or twice a week, or more than once or twice a week, reached approximately 60% in ELSA-EN compared to 25% in HAPIEE-PO. ELSA-EN participants were most likely to have no educational qualifications than those in Czech, Polish and Russian samples, which may reflect broader access to education during Communism in Central and Eastern Europe (CEE) (Tufis, 2010). There was an appreciable cross-country similarity in the preponderance of married or cohabitating participants. The larger proportion of widowers in ELSA-EN is due to age differences between the studies. Compared to the HAPIEE samples, ELSA-EN exhibited more favourable levels of self-rated health, yet a higher number of limitations in ADLs. The baseline prevalence of probable depression was close to double in HAPIEE-PO and HAPIEE-RU at 40% and 39%, respectively, than in ELSA-EN and HAPIEE-CZ where risk varied between 20 and 23%.

**Table 1:** Study characteristics of the ELSA and HAPIEE analytic samples.

Harmonised study variables^a^	ELSA-EN^b^ (N = 8519)		HAPIEE-CZ (N = 4908)		HAPIEE-PO (N = 6474)		HAPIEE-RU (N = 6180)	
	Mean or %	N	Mean or %	N	Mean or %	N	Mean or %	N
*Follow-up measures*								
CES-D 8 score (0–8)	1.6	8519	1.1	4908	2.2	6474	2.9	6180
*Baseline measures*								
Perceived neighbourhood social cohesion (PSC)								
High	30.7	2615	48.7	2390	35.6	2305	38.0	2349
Medium	26.7	2275	30.9	1517	27.7	1793	32.9	2033
Low	42.6	3629	20.4	1001	36.7	2376	29.1	1798
Age (years)	64.3	8519	58.4	4908	57.6	6474	58.2	6180
Female	55.3	4708	54.7	2685	51.6	3341	56.1	3467
Low control of life	15.8	1346	13.4	658	16.4	1062	15.9	983
Low control at home	8.5	724	17.9	879	17.3	1120	20.8	1285
Smoking status								
Never	36.2	3084	47.1	2312	40.9	2648	60.9	3764
Former	46.4	3953	29.4	1443	28.7	1858	13.5	834
Current	17.4	1482	23.5	1153	30.4	1968	25.6	1582
Drinking frequency								
Never	10.8	920	10.2	501	31.8	2059	14.6	902
Less than once a month	18.5	1576	25.9	1271	24.0	1554	39.3	2429
Once or several times a month	10.8	920	21.9	1075	20.9	1353	21.1	1304
Once or twice a week	31.1	2649	18.5	908	14.1	913	18.7	1156
More than once or twice a week	28.8	2453	23.5	1153	9.2	595	6.3	389
Educational attainment								
Higher education	23.7	2019	16.0	785	31.2	2020	30.2	1866
Intermediate	36.8	3135	74.1	3637	59.2	3833	60.4	3733
No qualifications	39.5	3365	9.9	486	9.6	621	9.4	581
Marital status								
Single	5.5	469	2.7	132	5.1	330	3.9	241
Married/cohabitating	66.8	5691	76.4	3750	78.0	5050	72.6	4487
Divorced/separated	10.8	920	12.2	599	6.7	434	9.9	612
Widowed	16.9	1440	8.7	427	10.2	660	13.6	840
Self-rated health								
Very good	21.2	1806	3.4	167	4.1	266	0.2	12
Good	35.3	3007	40.5	1988	33.6	2175	10.4	643
Fair	27.1	2310	47.1	2311	49.2	3185	68.7	4246
Bad or very bad	16.4	1396	9.0	442	13.1	848	20.7	1279
N of limitations in activities of daily living (0–5)	1.8	8519	1.1	4908	1.6	6474	1.3	6180
Probable depressive cases (CES-D 8 score ≥ 3^c^/CES-D 20 score ≥16^d^)	23.1	1965	20.2	991	40.7	2635	39.1	2416
CES-D 8 score (0–8)^c^/CES-D 20 score (0–60)^d^	1.5	8519	9.8	4908	11.1	6474	13.1	6180

a All estimates are averaged over the multiply imputed data sets.

b The ELSA estimates are also corrected for participant non-response at baseline.

c ELSA-specific measure based on the CES-D 8 score ranging from 0 to 8.

d HAPIEE-specific measure based on the CES-D 20 score ranging from 0 to 60.

### 3.2 Longitudinal associations between perceived neighbourhood social cohesion and depressive symptoms

Table 2 provides the country-specific results of multivariable negative binomial regression to assess the longitudinal association between PSC and depressive symptoms. The log count of the number of depressive symptoms increased in a dose-response manner for each tertile decrease in PSC after accounting for explanatory covariates in each sample, although the size and strength of associations varied between countries. Stepwise increases in symptoms from high to low PSC tertiles were found in all countries. However, differences in the expected log counts of symptoms between the high and medium PSC tertiles were modest and not statistically significant in ELSA-EN, HAPIEE-CZ and HAPIEE-PO. In HAPIEE-RU, however, the expected log counts of symptoms for adults with medium PSC was larger and statistically significant at 0.074 than for the referent. Risk differences between low and high PSC tertiles were strong in all countries, but varied in effect size. The expected differences in log counts were smallest in HAPIEE-RU at 0.087, then moderately larger in ELSA-EN and HAPIEE-CZ at 0.106 and 0.115. The risk difference among adults with low PSC was the most substantial in HAPIEE-PO at 0.203. For purposes of comparison, the subsequent path modelling results are reported for low vs. high PSC as these associations were strong in all countries.

**Table 2:** Depressive symptoms by tertiles of perceived neighbourhood social cohesion, ELSA and HAPIEE.

Negative binomial regression model^a^	ELSA-EN (N = 8519)			HAPIEE-CZ (N = 4908)			HAPIEE-PO (N = 6474)			HAPIEE-RU (N = 6180)		
	b	SE	P	b	SE	P	b	SE	P	b	SE	P
High	Reference			Reference			Reference			Reference		
Medium	0.003	0.037	0.942	0.027	0.050	0.589	0.027	0.033	0.416	0.074	0.023	0.002
Low	0.106	0.033	0.001	0.203	0.063	<0.001	0.115	0.030	<0.001	0.087	0.024	<0.001

a : All estimates are adjusted for age, gender, educational attainment, marital status, self-rated health, number of limitations in ADLs and probable depressive cases.

### 3.3 Pathway effects between low perceived neighbourhood social cohesion and depressive symptoms

Table 3 presents the country-specific unstandardized path coefficients relating low PSC to depressive symptoms, as envisaged by the path model in Fig. 1. Independent of all other relations in the model, the direct effects from low PSC to depressive symptoms remained strong and statistically significant in all countries. The higher expected log counts among adults with low PSC ranged from 0.083 in HAPIEE-RU to 0.187 in HAPIEE-CZ.

**Table 3:** Path coefficients relating low perceived social neighbourhood cohesion to depressive symptoms, ELSA and HAPIEE.

Path^a^		ELSA (N = 8519)				HAPIEE-CZ (N = 4908)			HAPIEE-PO (N = 6474)			HAPIEE-RU (N = 6180)		
from	to	b	SE	P-value	b		SE	P-value	b	SE	P-value	b	SE	P-value
Low PSC	Depressive symptoms^b^	0.097	0.033	0.003	0.187		0.052	<0.001	0.110	0.030	<0.001	0.083	0.024	0.001
Low PSC	Low control of life^c^	−0.272	0.071	<0.001	0.061		0.098	0.537	0.101	0.076	0.183	−0.244	0.073	0.001
Low PSC	Low control at home^c^	0.702	0.109	<0.001	0.623		0.106	<0.001	0.315	0.080	<0.001	0.866	0.090	<0.001
Low PSC	Current smoking status^c^	0.257	0.074	<0.001	0.263		0.087	0.003	0.124	0.064	0.050	0.087	0.072	0.225
Low PSC	Drinking frequency^c^	0.132	0.052	0.011	−0.055		0.070	0.433	0.161	0.053	0.002	0.004	0.056	0.946
Low control of life	Depressive symptoms^b^	0.168	0.032	<0.001	0.061		0.052	0.242	0.077	0.031	0.012	0.077	0.023	0.001
Low control at home	Depressive symptoms^b^	0.250	0.038	<0.001	0.283		0.055	<0.001	0.090	0.033	0.007	0.057	0.026	0.028
Current smoking	Depressive symptoms^b^	0.109	0.038	0.004	0.035		0.055	0.527	0.157	0.032	<0.001	0.081	0.030	0.008
Drinking frequency	Depressive symptoms^b^	−0.025	0.010	0.010	−0.009		0.017	0.602	0.000	0.011	0.978	−0.039	0.010	<0.001

a Path estimates from the independent exogenous variables to PSC and depressive symptoms are not shown for ease of interpretation.

b Path estimates are negative binomial regression coefficients, and refer to the difference in the expected log count of the number of depressive symptoms between the exposed group(s) and the reference group for each categorical or ordinal measure.

c Path estimates are logistic or ordered logistic regression coefficients, and denote the change in the predicted log odds or ordered log odds of the low PSC tertile being in the exposed group(s) for each categorical or ordinal measure.

Path estimates from low PSC to low control at home and current smoking were generally consistent between countries, and displayed only minor differences. The predicted log odds of low control at home for adults with low PSC were significantly higher compared to those with high PSC in all countries, but ranged from 0.315 in HAPIEE-PO to 0.866 in HAPIEE-RU. Adults with low PSC also had a greater log odds of being a current smoker than adults with high PSC, but this effect was smaller and weaker in HAPIEE-RU. Path estimates from low PSC to low control of life and drinking frequency exposed substantial cross-country differences. Adults with low PSC had a greater log odds of perceiving low control over their lives than those with high PSC in HAPIEE-CZ (0.061) and HAPIEE-PO (0.101), but these effects were statistically insignificant. Unexpectedly, adults with low PSC had statistically significant reduced log odds to perceive low control of life than the referents in ELSA-EN (−0.272) and HAPIEE-RU (−0.244). Lastly, the predicted ordered log odds of drinking alcohol to a higher frequency among adults with low PSC compared to the referents were sizeable and statistically significant in ELSA-EN (0.132) and HAPIEE-PO (0.161). Contrarily, the effects of low PSC on drinking behaviour were weak and inconsistent in HAPIEE-CZ and HAPIEE-RU.

Nonetheless, path relations from the considered mediators to depressive symptoms were predominantly congruent between countries. Perceiving low control of life, and at home, as well as current smoking behaviour prognosticated a higher number of depressive symptoms to a statistically significant degree in every country, except for HAPIEE-CZ. The weaker effects of low control of life and current smoking on depressive symptoms for Czech adults may reflect insufficient power because HAPIEE-CZ has the smallest sample size and the fewest number of depressive symptoms at follow-up. Unlike the harmful effects of the abovementioned mediators on depressive symptoms, a stepwise increase in drinking frequency strongly predicted fewer depressive symptoms in ELSA-EN and HAPIEE-RU, but again the effect was weaker in HAPIEE-CZ. In HAPIEE-PO, there was no risk difference in symptoms according to drinking frequency.

The country-specific decomposition of effects between low PSC and depressive symptoms are presented in Table 4. As reported earlier, the direct effects in each country remained strong after incorporating the psychological and health behavioural pathways. The total indirect effects, however, predicted higher expected log counts of depressive symptoms for low PSC adults in ELSA-EN (0.155) and HAPIEE-CZ (0.190) than in HAPIEE-PO (0.056) and HAPIEE-RU (0.038); suggesting that the examined pathways played a greater role on the PSC-depressive symptoms relationship in the two former countries. The total indirect effects were not only the smallest in HAPIEE-RU, but were weakly significant. The total effect between low PSC and depressive symptoms, as theorised by the path model, was therefore larger in ELSA-EN (0.252) and HAPIEE-CZ (0.377) than in HAPIEE-PO (0.165) and HAPIEE-RU (0.121).

**Table 4:** Decomposition of effects between low perceived neighbourhood social cohesion and depressive symptoms, ELSA and HAPIEE.

Pathways^a^	ELSA (N = 8519)			HAPIEE-CZ (N = 4908)			HAPIEE-PO (N = 6474)			HAPIEE-RU (N = 6180)		
	b	SE	P-value	b	SE	P-value	b	SE	P-value	b	SE	P-value
Direct effect	0.097	0.033	0.003	0.187	0.052	<0.001	0.110	0.030	<0.001	0.083	0.024	0.001
Total indirect effects	0.155	0.043	<0.001	0.190	0.048	<0.001	0.056	0.018	0.002	0.038	0.025	0.139
Total effect	0.252	0.051	<0.001	0.377	0.070	<0.001	0.165	0.034	<0.001	0.121	0.032	<0.001
Indirect effects via low control of life and low control at home	0.130	0.041	0.002	0.180	0.046	<0.001	0.036	0.014	0.012	0.031	0.025	0.213
Indirect effects via current smoking and drinking frequency	0.025	0.013	0.059	0.010	0.015	0.517	0.020	0.011	0.071	0.007	0.006	0.271
Specific indirect effects via												
Low control of life	−0.046	0.014	0.001	0.004	0.007	0.583	0.008	0.007	0.246	−0.019	0.008	0.020
Low control at home	0.175	0.038	<0.001	0.176	0.045	<0.001	0.028	0.013	0.025	0.049	0.023	0.034
Current smoking	0.028	0.013	0.029	0.009	0.015	0.537	0.019	0.011	0.069	0.007	0.006	0.268
Drinking frequency	−0.003	0.002	0.081	0.000	0.001	0.667	0.000	0.002	0.978	0.000	0.002	0.946

a Pathway estimates denote differences in the expected log counts of the number of depressive symptoms between the low and high PSC tertiles that are observed directly (direct effect), via all pathway variables (total indirect effect) and via hypothesised mediators (indirect effect). The total effect is the sum of the direct and the total indirect effect.

These differences aside, the total indirect effect between low PSC and depressive symptoms was notably attributable to the psychological pathway in each country, ranging from 64% in HAPIEE- PO (0.036/0.056) to 95% in HAPIEE-CZ (0.180/0.190). This was due to the strong specific indirect effects via low control at home across all countries, followed by low control of life which was statistically significant in ELSA-EN and HAPIEE-RU. The health behavioural pathway exerted a minor contribution to the total indirect effect as the specific effect via drinking frequency was negligible in all countries, and the effect via current smoking only appeared strong in ELSA-EN.

### 3.4 Sensitivity analyses

Restricting the path models to participants without depressive symptoms at baseline indicated that the prospective results were not biased by including these cases in the main analysis. The direct effects of low PSC to depressive symptoms remained strong, and the direction of associations were identical to those previously reported in both studies. Similarly, the indirect effects between low PSC and depressive symptoms predominantly occurred through the psychological, rather than the health behavioural, pathway (see Supplementary Tables S1-S3 for results). Repeating the multivariable regression and path modelling analyses on a subset of ELSA participants aged 50–69 years (n = 5913) produced similar results as those for the entire analytic sample, and indicated that the comparative analyses were not hindered by the different age profiles in ELSA (50 + years) and HAPIEE (45–69 years) (Supplementary Tables S4-S6). Additional path models in the HAPIEE cohorts, which tested a health behavioural pathway via drinking volume and binge drinking episodes instead of drinking frequency, also showed very weak indirect effects between social cohesion and depressive symptoms through these other aspects of drinking behaviour (Supplementary Tables S7-S10).

## 4 Discussion

As a cultural aspect of the macro-level environment, social cohesion is undoubtedly influenced by governance, socio-economic and public policies (Berkman et al., 2000). This is made evident by levels of social cohesion which are markedly higher in England than in CEE countries (Anderson and Unzicker, 2014; Kääriäinen and Lehtonen, 2006; Poortinga, 2006). As social cohesion is known to suffer during economic and employment crises (Anderson and Unzicker, 2014), the loss of security and collapse of social institutions stemming from the post-communist transition weakened communal norms and social ties in CEE (Berkman et al., 2000; Bobak et al., 1998). Furthermore, these societal disruptions are thought to have elevated not only rates of mental illness (Abbott and Sapsford, 2006; Anderson and Unzicker, 2014), but led to upswings in mortality (Marmot and Bobak, 2005) in these countries. Cross-cultural studies that assess whether associations between social cohesion and risk of depressive disorders hold in populations with dissimilar histories, norms and values are largely absent. One may expect differences between the post-socialist welfare states of CEE and a liberal welfare state, such as England (Kääriäinen and Lehtonen, 2006). We address this uncertainty by showing that the prospective association between perceived neighbourhood social cohesion and elevated depressive symptoms was remarkably similar between older adults from diverse parts of Europe during the first decade of the 2000s.

To our knowledge, this is the first prospective study comparing the relations between social cohesion, or other aspects of cognitive social capital, and depressive symptoms between CEE with Western European countries. Cross-sectional evidence of country differences, however, has emerged in recent years (Hsieh, 2015; Karhina et al., 2016). A study comparing the role of low perceived neighbourhood safety, an oft-used marker of cognitive social capital, and elevated depressive symptoms between adults living in Sweden and Ukraine during 2003–2004 found strong associations for both genders in Sweden, but for women only in Ukraine (Karhina et al., 2016). Although our study was limited to Europe, an investigation between older adults in China and Russia – two countries that experienced decades of economic reforms with much worse social and health consequences for Russians – found that low perceived neighbourhood safety, but not low trust in neighbours, was associated with a higher number of depressive symptoms to the same effect in both countries (Hsieh, 2015).

There is a premise that low cognitive social capital is more harmful for individuals living in communities characterised by high social capital because these individuals face greater forms of social exclusion than if they resided in low social capital communities (Kawachi et al., 2004). While the lack of association among Ukrainian men (Karhina et al., 2016) may indicate such an interaction between the individual and contextual effect of cognitive social capital; our findings, and those by Hsieh (2015), found that individual effects of social cohesion on depressive symptoms were consistent despite contextual country differences. Literature reviews relating social capital to depressive and other common mental disorders have shown that most European evidence is disproportionately based on Western and Northern countries, as no review incorporated studies from CEE (De Silva et al., 2005; Ehsan and De Silva, 2015; Julien et al., 2012). Therefore, it is difficult to establish the extent to which associations should be consistent between the countries included in our analysis. Our study, in addition to the two discussed above, relied on perceived neighbourhood characteristics at the individual level to evaluate differences between countries. As the country context is theorised to influence social cohesion of individuals and communities, a rigorous exploration of potential interactions between individual and neighbourhood social cohesion on depressive symptoms requires a multi-level framework (Kawachi et al., 2004). Due to the lack of geographical data access in ELSA and HAPIEE, an outstanding study limitation is that we were unable to perform a comparative analysis of individuals nested in neighbourhoods using a multilevel framework.

Most studies on this topic are cross-sectional, which preclude the assessment of pathways (De Silva et al., 2005; Ehsan and De Silva, 2015; Julien et al., 2012), Moreover, longitudinal pathway analyses have primarily tested social network and social support mechanisms (Choi et al., 2015; Stafford et al., 2011) (Berkman et al., 2000; Blair et al., 2014). Although low social cohesion may increase the risk of depressive disorders via the reduction of positive psychological states (e.g., a sense of belonging, efficacy and security) and protection against the psychological harm of daily stressors, and also by curtailing unhealthy behaviours through normative guidance and control (Berkman et al., 2000; Blair et al., 2014; Kawachi and Berkman, 2001), these pathways remain uncertain. This analysis provides empirical support for a psychological, rather than a health behavioural, pathway. The consistency of pathway effects between four distinct ageing populations support the accuracy of these results. Sensitivity analyses confirmed that the longitudinal relations hypothesised by our path model were not biased by depressed individuals at baseline, who may have reported lower levels of social cohesion and self-efficacy, and more adverse health behaviours.

Nonetheless, our prospective study has several limitations. Firstly, our path model was based on a 2 to 3-year follow-up, which restricted us to a half-longitudinal design as both the exposure and mediators were measured at baseline. Due to the lack of further follow-up studies in HAPIEE, we could not carry out a comparative analysis over a longer time period to strengthen the prospective nature of our findings. Secondly, baseline and follow-up response rates of 59–69% and 63–77%, respectively, limits the representativeness of the target study populations in ELSA and HAPIEE. Therefore, our findings cannot be extrapolated to the general older population in England, nor to urban-dwelling older populations in the Czech Republic, Poland and Russia. This leads to the third limitation of our study. An ELSA technical report indicated that 25.6% of the cohort who participated in the 2002/3 and 2004/5 assessments, from which the ELSA analytic sample was drawn, lived in rural areas (Scholes et al., 2009). We were unfortunately unable to exclude rural-dwelling adults from the ELSA sample to maximise comparability with the urban-based HAPIEE samples because we were not granted access to ELSA data on urban-rural residence. Given that UK-based population studies suggest that rural-dwelling adults experience greater levels of social cohesion (Brook Lyndhurst Ltd, 2010) and lower rates of common mental disorders (Weich et al., 2006), the presence of rural-dwelling adults in the ELSA sample may have obscured differences in the relations between social cohesion and depressive symptoms between English and Central/Eastern European samples. Fourthly, although our aim was to test pathways that were overlooked in previous studies, social network and social support pathways remain important. A comprehensive path model would have tested multiple pathways simultaneously, but data on social networks and social support were not harmonizable across ELSA and HAPIEE. Lastly, the data harmonization precluded the use of other data that were collected on perceived neighbourhood social cohesion on the one hand, and self-efficacy on the other hand, due to lack of comparability across studies. Evaluating social cohesion and self-efficacy, based on two items respectively, may have oversimplified the complexity of these constructs and affected the accuracy of our findings. As the two-item PSC score for ELSA (2–14) and HAPIEE (2–10) were on different scales and not normally distributed, we harmonised the data by collapsing scores into country-specific tertiles. While this led to further loss of information, the tertiles allowed us to compare within-country associations between PSC and depressive symptoms as these groupings corresponded to different exposure values in each country sample. Despite these limitations, the decisions made toward the data harmonization were essential to achieve inferential equivalence for the comparative analyses (Fortier et al., 2016).

## 5 Conclusion

The effects of low perceived neighbourhood social cohesion on elevated depressive symptoms were strikingly congruent between older adults in England and the Czech Republic, Russia and Poland. The role of psychological and health behavioural markers that were tested as explanatory pathways relating low cohesion to heightened depressive symptoms were also remarkably synonymous between these older populations. Indirect effects through the psychological pathway were substantial and explained most of the mediating mechanisms hypothesised in our study. The health behavioural pathway, on the other hand, played a negligible role in this longitudinal relationship.

## Declaration of interest

The authors declare that there are no conflicts of interest.
