# Supplementary material for: Congruent relations between perceived neighbourhood social cohesion and depressive symptoms among older European adults: An East-West analysis
Source: Soc Sci Med. 2019 Sep;237:112454. doi: 10.1016/j.socscimed.2019.112454 (PMC6728599; doi:10.1016/j.socscimed.2019.112454)

**Title:** Congruent relations between perceived neighbourhood social cohesion and depressive symptoms among older European adults: An East-West analysis

**Supplementary Tables and Figures**

[Table S1 Depressive symptoms by tertiles of perceived neighbourhood social cohesion among non-cases of probable depression at baseline, ELSA and HAPIEE 2](#_Toc14690002)

[Table S2 Path coefficients relating low perceived neighbourhood social cohesion to depressive symptoms among non-cases of probable depression at baseline, ELSA and HAPIEE 2](#_Toc14690003)

[Table S3 Decomposition of effects between low perceived neighbourhood social cohesion and depressive symptoms among non-cases of probable depression at baseline, ELSA and HAPIEE 3](#_Toc14690004)

[Table S4 Depressive symptoms by tertiles of perceived neighbourhood social cohesion among adults aged 50-69 years, ELSA 4](#_Toc14690005)

[Table S5 Path coefficients relating low perceived neighbourhood social cohesion to depressive symptoms among adults aged 50-69 years, ELSA 4](#_Toc14690006)

[Table S6 Decomposition of effects between low perceived neighbourhood social cohesion and depressive symptoms among adults aged 50-69 years, ELSA 4](#_Toc14690007)

[Table S7 Path coefficients relating low perceived neighbourhood social cohesion to depressive symptoms with alternate pathway via annual drinking volume, HAPIEE 5](#_Toc14690008)

[Table S8 Decomposition of effects between low perceived neighbourhood social cohesion and depressive symptoms with alternate pathway via annual drinking volume, HAPIEE 6](#_Toc14690009)

[Table S9 Path coefficients relating low perceived neighbourhood social cohesion to depressive symptoms with alternate pathway via binge drinking frequency, HAPIEE 7](#_Toc14690010)

[Table S10 Decomposition of effects between low perceived neighbourhood social cohesion and depressive symptoms with alternate pathway via binge drinking frequency, HAPIEE 8](#_Toc14690011)

[Figure S1 Selection diagram of the analytic samples, ELSA and HAPIEE 9](#_Toc14690012)

## Table S1 Depressive symptoms by tertiles of perceived neighbourhood social cohesion among non-cases of probable depression at baseline, ELSA and HAPIEE

| **Negative binomial regression model^a^** | **ELSA-EN**  **(N=6,516)** | | | **HAPIEE-CZ**  **(N=3,868)** | | | **HAPIEE-PO**  **(N=4,649)** | | | **HAPIEE-RU**  **(N=4,534)** | | |
| --- | --- | --- | --- | --- | --- | --- | --- | --- | --- | --- | --- | --- |
|  | **b** | **SE** | **P** | **b** | **SE** | **P** | **b** | **SE** | **P** | **b** | **SE** | **P** |
| High | Reference | | | Reference | | | Reference | | | Reference | | |
| Medium | 0.010 | 0.049 | 0.841 | 0.035 | 0.060 | 0.561 | 0.035 | 0.043 | 0.416 | 0.108 | 0.029 | <0.001 |
| Low | 0.130 | 0.045 | 0.004 | 0.215 | 0.065 | 0.001 | 0.133 | 0.039 | 0.001 | 0.078 | 0.033 | 0.019 |

a: All estimates are adjusted for age, gender, educational attainment, marital status, self-rated health and number of limitations in ADLs.

## Table S2 Path coefficients relating low perceived neighbourhood social cohesion to depressive symptoms among non-cases of probable depression at baseline, ELSA and HAPIEE

| **Path^a^** | | **ELSA-EN**  **(N=6,516)** | | | **HAPIEE-CZ**  **(N=3,868)** | | | **HAPIEE-PO**  **(N=4,649)** | | | | **HAPIEE-RU**  **(N=4,534)** | | | |
| --- | --- | --- | --- | --- | --- | --- | --- | --- | --- | --- | --- | --- | --- | --- | --- |
| **from** | **to** | **b** | **SE** | **P-value** | **b** | **SE** | **P-value** | **b** | **SE** | **P-value** | **b** | | **SE** | **P-value** |  |
| Low PSC | Depressive symptoms**^b^** | 0.127 | 0.045 | 0.005 | 0.199 | 0.065 | 0.002 | 0.132 | 0.039 | 0.006 | 0.080 | | 0.033 | 0.016 |  |
| Low PSC | Low control of life**^c^** | -0.465 | 0.085 | <0.001 | 0.115 | 0.115 | 0.318 | 0.073 | 0.095 | 0.437 | -0.336 | | 0.092 | <0.001 |  |
| Low PSC | Low control at home**^c^** | 0.686 | 0.155 | <0.001 | 0.538 | 0.133 | <0.001 | 0.079 | 0.104 | 0.447 | 0.710 | | 0.115 | <0.001 |  |
| Low PSC | Current smoking status**^c^** | 0.333 | 0.87 | <0.001 | 0.226 | 0.101 | 0.024 | 0.072 | 0.075 | 0.343 | 0.085 | | 0.084 | 0.313 |  |
| Low PSC | Drinking frequency**^c^** | 0.157 | 0.060 | 0.009 | 0.049 | 0.081 | 0.546 | 0.256 | 0.063 | <0.001 | 0.087 | | 0.068 | 0.199 |  |
| Low control of life | Depressive symptoms**^b^** | 0.240 | 0.048 | <0.001 | -0.006 | 0.067 | 0.928 | 0.075 | 0.044 | 0.090 | 0.105 | | 0.032 | 0.001 |  |
| Low control at home | Depressive symptoms**^b^** | 0.272 | 0.074 | <0.001 | 0.301 | 0.072 | <0.001 | 0.103 | 0.051 | 0.041 | 0.077 | | 0.036 | 0.032 |  |
| Current smoking | Depressive symptoms**^b^** | 0.105 | 0.040 | 0.050 | -0.004 | 0.066 | 0.947 | 0.173 | 0.042 | <0.001 | 0.077 | | 0.039 | 0.049 |  |
| Drinking frequency | Depressive symptoms**^b^** | -0.036 | 0.014 | 0.010 | -0.006 | 0.021 | 0.766 | -0.015 | 0.015 | 0.325 | -0.031 | | 0.013 | 0.018 |  |

a: Path estimates from the independent exogenous variables to PSC and depressive symptoms are not shown for ease of interpretation.

b: Path estimates are negative binomial regression coefficients, and refer to the difference in the expected log count of the number of depressive symptoms between the exposed group(s) and the reference group for each categorical or ordinal measure.

c: Path estimates are logistic or ordered logistic regression coefficients, and denote the change in the predicted log odds or ordered log odds of the low PSC tertile being in the exposed group(s) for each categorical or ordinal measure.

## Table S3 Decomposition of effects between low perceived neighbourhood social cohesion and depressive symptoms among non-cases of probable depression at baseline, ELSA and HAPIEE

| **Pathway^a^** | **ELSA-EN**  **(N=6,516)** | | | | **HAPIEE-CZ**  **(N=3,868)** | | | | **HAPIEE-PO**  **(N=4,649)** | | | **HAPIEE-RU**  **(N=4,534)** | | |
| --- | --- | --- | --- | --- | --- | --- | --- | --- | --- | --- | --- | --- | --- | --- |
|  | **b** | **SE** | **P-value** | **b** | | **SE** | **P-value** | **b** | | **SE** | **P-value** | **b** | **SE** | **P-value** |
| Direct effect | 0.127 | 0.045 | 0.005 | 0.199 | | 0.065 | 0.002 | 0.132 | | 0.039 | 0.006 | 0.080 | 0.033 | 0.016 |
| Total indirect effects | 0.104 | 0.078 | 0.181 | 0.160 | | 0.058 | 0.006 | 0.022 | | 0.020 | 0.262 | 0.023 | 0.031 | 0.456 |
| Total effect | 0.231 | 0.086 | 0.007 | 0.359 | | 0.087 | <0.001 | 0.154 | | 0.044 | <0.001 | 0.103 | 0.043 | 0.016 |
| Indirect effects via low control of life and low control at home | 0.075 | 0.074 | 0.313 | 0.161 | | 0.056 | 0.004 | 0.014 | | 0.014 | 0.323 | 0.019 | 0.030 | <0.001 |
| Indirect effects via current smoking and drinking frequency | 0.029 | 0.020 | 0.149 | -0.001 | | 0.015 | 0.932 | 0.009 | | 0.014 | 0.532 | 0.004 | 0.007 | 0.001 |
| Specific indirect effects via |  |  |  |  | |  |  |  | |  |  |  |  |  |
| Low control of life | -0.112 | 0.029 | <0.001 | -0.001 | | 0.008 | 0.928 | 0.006 | | 0.008 | 0.488 | -0.035 | 0.015 | 0.015 |
| Low control at home | 0.187 | 0.067 | 0.005 | 0.162 | | 0.056 | 0.004 | 0.008 | | 0.011 | 0.474 | 0.055 | 0.027 | 0.046 |
| Current smoking | 0.035 | 0.020 | 0.082 | -0.001 | | 0.015 | 0.947 | 0.012 | | 0.013 | 0.357 | 0.007 | 0.007 | 0.367 |
| Drinking frequency | -0.006 | 0.003 | 0.072 | 0.000 | | 0.001 | 0.800 | -0.004 | | 0.004 | 0.340 | -0.003 | 0.002 | 0.265 |

a: Pathway estimates denote differences in the expected log counts of the number of depressive symptoms between the low and high PSC tertiles that are observed directly (direct effect), via all pathway variables (total indirect effect) and via hypothesised mediators (indirect effect). The total effect is the sum of the direct and the total indirect effect.

## Table S4 Depressive symptoms by tertiles of perceived neighbourhood social cohesion among adults aged 50-69 years, ELSA

| **Negative binomial regression model^a^** | **ELSA-EN (N=5,913)** | | |
| --- | --- | --- | --- |
|  | **b** | **SE** | **P** |
| High | Reference | | |
| Medium | -0.014 | 0.049 | 0.783 |
| Low | 0.137 | 0.042 | 0.001 |

a: All estimates are adjusted for age, gender, educational attainment, marital status, self-rated health, number of limitations in ADLs and probable depressive cases.

## Table S5 Path coefficients relating low perceived neighbourhood social cohesion to depressive symptoms among adults aged 50-69 years, ELSA

| **Path^a^** | | **ELSA-EN (N=5,913)** | | |
| --- | --- | --- | --- | --- |
| **from** | **to** | **b** | **SE** | **P-value** |
| Low PSC | Depressive symptoms**^b^** | 0.132 | 0.042 | 0.002 |
| Low PSC | Low control of life**^c^** | -0.261 | 0.091 | 0.004 |
| Low PSC | Low control at home**^c^** | 0.739 | 0.140 | <0.001 |
| Low PSC | Current smoking status**^c^** | 0.142 | 0.085 | 0.097 |
| Low PSC | Drinking frequency**^c^** | 0.043 | 0.065 | 0.508 |
| Low control of life | Depressive symptoms**^b^** | 0.218 | 0.042 | <0.001 |
| Low control at home | Depressive symptoms**^b^** | 0.280 | 0.048 | <0.001 |
| Current smoking | Depressive symptoms**^b^** | 0.091 | 0.045 | 0.043 |
| Drinking frequency | Depressive symptoms**^b^** | -0.043 | 0.013 | 0.001 |

## Table S6 Decomposition of effects between low perceived neighbourhood social cohesion and depressive symptoms among adults aged 50-69 years, ELSA

| **Pathway^a^** | **ELSA-EN (N=5,913)** | | |
| --- | --- | --- | --- |
|  | **b** | **SE** | **P-value** |
| Direct effect | 0.132 | 0.042 | 0.002 |
| Total indirect effects | 0.161 | 0.057 | 0.005 |
| Total effect | 0.293 | 0.070 | <0.001 |
| Indirect effects via low control of life and low control at home | 0.150 | 0.056 | 0.008 |
| Indirect effects via current smoking and drinking frequency | 0.011 | 0.011 | 0.309 |
| Specific indirect effects via |  |  |  |
| Low control of life | -0.057 | 0.022 | 0.010 |
| Low control at home | 0.207 | 0.050 | <0.001 |
| Current smoking | 0.013 | 0.010 | 0.206 |
| Drinking frequency | -0.002 | 0.003 | 0.519 |

## Table S7 Path coefficients relating low perceived neighbourhood social cohesion to depressive symptoms with alternate pathway via annual drinking volume, HAPIEE

| **Path^a^** | | **HAPIEE-CZ**  **(N=4,908)** | | | **HAPIEE-PO**  **(N=6,474)** | | | | **HAPIEE-RU**  **(N=6,180)** | | | |
| --- | --- | --- | --- | --- | --- | --- | --- | --- | --- | --- | --- | --- |
| **from** | **to** | **b** | **SE** | **P-value** | **b** | **SE** | **P-value** | **b** | | **SE** | **P-value** |  |
| Low PSC | Depressive symptoms**^b^** | 0.190 | 0.052 | <0.001 | 0.110 | 0.030 | <0.001 | 0.085 | | 0.024 | <0.001 |  |
| Low PSC | Low control of life**^c^** | 0.058 | 0.098 | 0.558 | 0.097 | 0.076 | 0.200 | -0.244 | | 0.073 | 0.001 |  |
| Low PSC | Low control at home**^c^** | 0.622 | 0.106 | <0.001 | 0.317 | 0.080 | <0.001 | 0.867 | | 0.090 | <0.001 |  |
| Low PSC | Current smoking status**^c^** | 0.263 | 0.087 | 0.003 | 0.124 | 0.064 | 0.050 | 0.087 | | 0.072 | 0.225 |  |
| Low PSC | Drinking volume**^c^** | -0.058 | 0.068 | 0.388 | 0.125 | 0.053 | 0.019 | -0.012 | | 0.055 | 0.827 |  |
| Low control of life | Depressive symptoms**^b^** | 0.065 | 0.052 | 0.215 | 0.079 | 0.031 | 0.011 | 0.077 | | 0.023 | 0.001 |  |
| Low control at home | Depressive symptoms**^b^** | 0.284 | 0.055 | <0.001 | 0.090 | 0.033 | 0.007 | 0.057 | | 0.026 | 0.027 |  |
| Current smoking | Depressive symptoms**^b^** | 0.032 | 0.055 | 0.559 | 0.153 | 0.032 | <0.001 | 0.080 | | 0.030 | 0.009 |  |
| Drinking volume | Depressive symptoms**^b^** | 0.002 | 0.015 | 0.905 | 0.005 | 0.011 | 0.617 | -0.019 | | 0.008 | 0.022 |  |

a: Path estimates from the independent exogenous variables to PSC and depressive symptoms are not shown for ease of interpretation.

b: Path estimates are negative binomial regression coefficients, and refer to the difference in the expected log count of the number of depressive symptoms between the exposed group(s) and the reference group for each categorical or ordinal measure.

c: Path estimates are logistic or ordered logistic regression coefficients, and denote the change in the predicted log odds or ordered log odds of the low PSC tertile being in the exposed group(s) for each categorical or ordinal measure.

## Table S8 Decomposition of effects between low perceived neighbourhood social cohesion and depressive symptoms with alternate pathway via annual drinking volume, HAPIEE

| **Pathway^a^** | **HAPIEE-CZ**  **(N=4,908)** | | | | **HAPIEE-PO**  **(N=6,474)** | | | **HAPIEE-RU**  **(N=6,180)** | | | |  |
| --- | --- | --- | --- | --- | --- | --- | --- | --- | --- | --- | --- | --- |
|  | **b** | **SE** | **P-value** | **b** | | **SE** | **P-value** | | **b** | **SE** | **P-value** | |
| Direct effect | 0.190 | 0.052 | <0.001 | 0.110 | | 0.030 | <0.001 | | 0.085 | 0.024 | <0.001 | |
| Total indirect effects | 0.189 | 0.048 | <0.001 | 0.056 | | 0.018 | 0.002 | | 0.038 | 0.025 | 0.135 | |
| Total effect | 0.378 | 0.070 | <0.001 | 0.166 | | 0.035 | <0.001 | | 0.123 | 0.033 | <0.001 | |
| Indirect effects via low control of life and low control at home | 0.180 | 0.046 | <0.001 | 0.036 | | 0.014 | 0.012 | | 0.031 | 0.025 | 0.211 | |
| Indirect effects via current smoking and drinking volume | 0.008 | 0.015 | 0.575 | 0.020 | | 0.011 | 0.062 | | 0.007 | 0.006 | 0.248 | |
| Specific indirect effects via |  |  |  |  | |  |  | |  |  |  | |
| Low control of life | 0.004 | 0.007 | 0.595 | 0.008 | | 0.007 | 0.258 | | -0.019 | 0.008 | 0.020 | |
| Low control at home | 0.177 | 0.045 | <0.001 | 0.028 | | 0.013 | 0.025 | | 0.049 | 0.023 | 0.033 | |
| Current smoking | 0.008 | 0.015 | 0.567 | 0.019 | | 0.011 | 0.070 | | 0.007 | 0.006 | 0.270 | |
| Drinking volume | 0.000 | 0.001 | 0.905 | 0.001 | | 0.001 | 0.624 | | 0.000 | 0.001 | 0.827 | |

a: Pathway estimates denote differences in the expected log counts of the number of depressive symptoms between the low and high PSC tertiles that are observed directly (direct effect), via all pathway variables (total indirect effect) and via hypothesised mediators (indirect effect). The total effect is the sum of the direct and the total indirect effect.

## Table S9 Path coefficients relating low perceived neighbourhood social cohesion to depressive symptoms with alternate pathway via binge drinking frequency, HAPIEE

| **Path^a^** | | **HAPIEE-CZ**  **(N=4,908)** | | | **HAPIEE-PO**  **(N=6,474)** | | | | **HAPIEE-RU**  **(N=6,180)** | | | |
| --- | --- | --- | --- | --- | --- | --- | --- | --- | --- | --- | --- | --- |
| **from** | **to** | **b** | **SE** | **P-value** | **b** | **SE** | **P-value** | **b** | | **SE** | **P-value** |  |
| Low PSC | Depressive symptoms**^b^** | 0.190 | 0.052 | <0.001 | 0.110 | 0.030 | <0.001 | 0.086 | | 0.024 | <0.001 |  |
| Low PSC | Low control of life**^c^** | 0.058 | 0.098 | 0.558 | 0.097 | 0.076 | 0.200 | -0.244 | | 0.073 | 0.001 |  |
| Low PSC | Low control at home**^c^** | 0.622 | 0.106 | <0.001 | 0.317 | 0.080 | <0.001 | 0.867 | | 0.090 | <0.001 |  |
| Low PSC | Current smoking status**^c^** | 0.263 | 0.087 | 0.003 | 0.124 | 0.064 | 0.050 | 0.087 | | 0.072 | 0.225 |  |
| Low PSC | Binge drinking**^c^** | 0.244 | 0.084 | 0.004 | 0.181 | 0.080 | 0.023 | 0.105 | | 0.075 | 0.162 |  |
| Low control of life | Depressive symptoms**^b^** | 0.064 | 0.052 | 0.219 | 0.079 | 0.031 | 0.011 | 0.079 | | 0.023 | 0.001 |  |
| Low control at home | Depressive symptoms**^b^** | 0.284 | 0.055 | <0.001 | 0.089 | 0.033 | 0.007 | 0.057 | | 0.026 | 0.028 |  |
| Current smoking | Depressive symptoms**^b^** | 0.026 | 0.055 | 0.629 | 0.149 | 0.032 | <0.001 | 0.075 | | 0.030 | 0.013 |  |
| Binge drinking | Depressive symptoms**^b^** | 0.023 | 0.028 | 0.413 | 0.037 | 0.022 | 0.097 | -0.015 | | 0.013 | 0.267 |  |

a: Path estimates from the independent exogenous variables to PSC and depressive symptoms are not shown for ease of interpretation.

b: Path estimates are negative binomial regression coefficients, and refer to the difference in the expected log count of the number of depressive symptoms between the exposed group(s) and the reference group for each categorical or ordinal measure.

c: Path estimates are logistic or ordered logistic regression coefficients, and denote the change in the predicted log odds or ordered log odds of the low PSC tertile being in the exposed group(s) for each categorical or ordinal measure.

## Table S10 Decomposition of effects between low perceived neighbourhood social cohesion and depressive symptoms with alternate pathway via binge drinking frequency, HAPIEE

| **Pathway^a^** | **HAPIEE-CZ**  **(N=4,908)** | | | | **HAPIEE-PO**  **(N=6,474)** | | | **HAPIEE-RU**  **(N=6,180)** | | | |  |
| --- | --- | --- | --- | --- | --- | --- | --- | --- | --- | --- | --- | --- |
|  | **b** | **SE** | **P-value** | **b** | | **SE** | **P-value** | | **b** | **SE** | **P-value** | |
| Direct effect | 0.190 | 0.052 | <0.001 | 0.110 | | 0.030 | <0.001 | | 0.086 | 0.024 | <0.001 | |
| Total indirect effects | 0.193 | 0.048 | <0.001 | 0.061 | | 0.019 | 0.001 | | 0.035 | 0.025 | 0.165 | |
| Total effect | 0.383 | 0.070 | <0.001 | 0.171 | | 0.035 | <0.001 | | 0.121 | 0.032 | <0.001 | |
| Indirect effects via low control of life and low control at home | 0.181 | 0.046 | <0.001 | 0.036 | | 0.014 | 0.013 | | 0.030 | 0.025 | 0.221 | |
| Indirect effects via current smoking and binge drinking | 0.013 | 0.016 | 0.417 | 0.025 | | 0.012 | 0.031 | | 0.005 | 0.006 | 0.392 | |
| Specific indirect effects via |  |  |  |  | |  |  | |  |  |  | |
| Low control of life | 0.004 | 0.007 | 0.595 | 0.008 | | 0.007 | 0.259 | | -0.019 | 0.008 | 0.018 | |
| Low control at home | 0.177 | 0.045 | <0.001 | 0.028 | | 0.013 | 0.026 | | 0.049 | 0.023 | 0.034 | |
| Current smoking | 0.007 | 0.015 | 0.634 | 0.019 | | 0.010 | 0.070 | | 0.007 | 0.006 | 0.274 | |
| Binge drinking | 0.006 | 0.007 | 0.435 | 0.007 | | 0.005 | 0.188 | | -0.002 | 0.002 | 0.381 | |

a: Pathway estimates denote differences in the expected log counts of the number of depressive symptoms between the low and high PSC tertiles that are observed directly (direct effect), via all pathway variables (total indirect effect) and via hypothesised mediators (indirect effect). The total effect is the sum of the direct and the total indirect effect.

## Figure S1 Selection diagram of the analytic samples, ELSA and HAPIEE


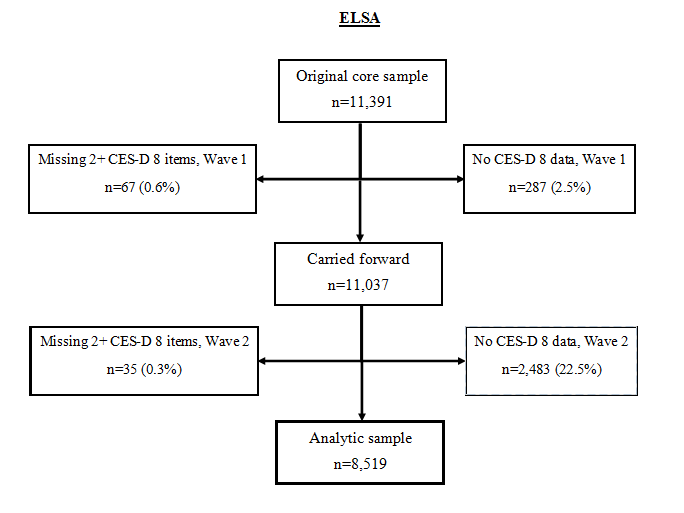


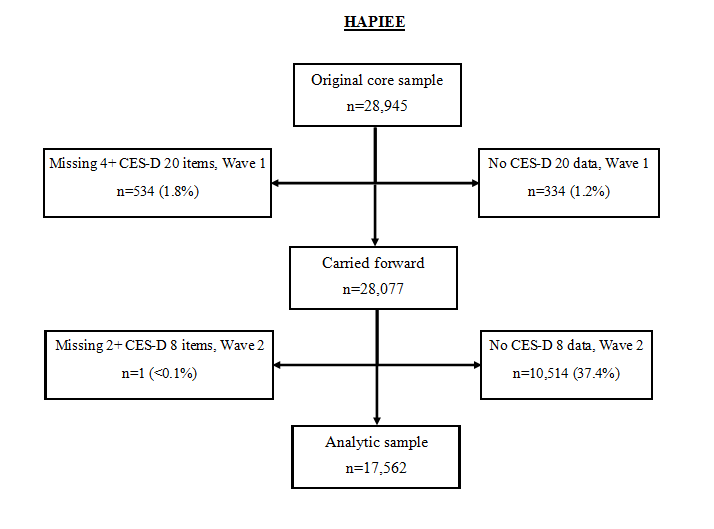

Supplement: Multimedia component 1 [file mmc1.docx]
